# Citizens and scientists collect comparable oceanographic data: measurements of ocean transparency from the Secchi Disk study and science programmes

**DOI:** 10.1038/s41598-021-95029-z

**Published:** 2021-07-29

**Authors:** Richard R. Kirby, Gregory Beaugrand, Loick Kleparski, Susie Goodall, Samantha Lavender

**Affiliations:** 1 The Secchi Disk Foundation, Kiln Cottage, Gnaton, Yealmpton, PL8 2HU Devon UK; 2 UMR 8187 - LOG - Laboratoire d’Océanologie et de Géosciences, CNRS, Univ. Lille, Univ. Littoral Côte d’Opale, 59000 Lille, France; 3 14 Osprey Close, Southampton, SO40 4XJ Hampshire UK; 4 Pixalytics Ltd, Plymouth Science Park, 1 Davy Rd, Plymouth, PL6 8BX Devon UK; 5 The Marine Biological Association, Citadel Hill, The Hoe, Plymouth, PL1 2PB Devon UK

**Keywords:** Marine biology, Environmental impact

## Abstract

Marine phytoplankton accounts for approximately 50% of all photosynthesis on Earth, underpins the marine food chain and plays a central role in the Earth’s biogeochemical cycles and climate. In situ measurements of ocean transparency can be used to estimate phytoplankton biomass. The scale and challenging conditions of the ocean make it a difficult environment for in situ studies, however. Here, we show that citizen scientists (seafarers) using a simple white Secchi Disk can collect ocean transparency data to complement formal scientific efforts using similar equipment. Citizen scientist data can therefore help understand current climate-driven changes in phytoplankton biomass at a global scale.

## Introduction

The ocean is a difficult environment to access for in situ study due to its scale, remoteness and challenging conditions. Although ocean science research is vital for our sustainable future^1^, scientific research lags current, climate-driven ocean changes^2^. The ocean’s phytoplankton account for approximately 50% of all photosynthesis on Earth^3,4^ and temporal and spatial changes in the phytoplankton can influence marine productivity^5^, weather^6^ and climate^7,8^. Monitoring the phytoplankton is therefore essential as an early indicator of regional and global ecosystem change^9–12^.

Around 44% of the human population lives within 150 km of the coast^13^ and a number go to sea for work and recreation. The seafaring public often visits the same locations, whether as sailors on short day trips, commercial fishermen accessing fishing grounds, or offshore yachtsmen/women whose passages follow common routes dictated by the season, prevailing winds and currents^14^. Therefore, the seafaring public provides an opportunity to collect oceanographic data over varying spatial and temporal scales to contribute to scientific efforts and many now participate in marine citizen science^15,16^.

The global Secchi Disk study (http://www.secchidisk.org)^17^ engages seafarers to use a Secchi Disk^18^ to collect in situ data on ocean transparency that can be used to estimate phytoplankton biomass^19^. The Secchi Disk study uses a simple, 30 cm diameter white Secchi Disk that is weighted and attached to a tape-measure, and lowered vertically into the water from a boat’s side. The depth (m) below the surface when the Secchi Disk disappears from sight is the Secchi depth (*Z*_SD_), which measures ocean transparency. When the bathymetry is > 25 m depth and the distance > 1 km from shore, the primary influence upon ocean transparency is phytoplankton pigments and their breakdown products and therefore, *Z*_SD_ estimates phytoplankton biomass in the water column; re-suspended sediments and dissolved organic matter from rivers further reduce transparency and introduce optical errors in shallower water and closer inshore^12^.

Marine scientists have used Secchi Disks to measure ocean transparency since 1865^20^ and archives of *Z*_SD_ represent one of the longest-running, spatially extensive global marine datasets^21^. Recently, the Secchi Disk has fallen from widespread use among marine scientists^22^ due to spectrophotometric determination of chlorophyll *a*^12^ and satellite measures of ocean colour^23^. To re-establish the database of *Z*_SD_ measurements and facilitate contemporary *Z*_SD_ comparisons to historical data, the citizen science Secchi Disk study began in 2013^17^.

Measuring in situ data at sea from a small boat is challenging. For citizen science efforts of any type to be useful it is essential to ensure and assess data quality^24,25^, and this was outlined recently in the ninth of the ten principles of citizen science outlined by the European Citizen Science Association (ECSA)^26^. Marine and terrestrial, observational, citizen science studies have assessed data quality and shown that citizens can collect reliable observational data when given good instructions^27–29^. We have already shown that *Z*_SD_ data collected by citizens agrees with satellite measures of chlorophyll *a* and satellite *Z*_SD_ estimates^17^. But, does *Z*_SD_ data collected by citizens of varying backgrounds using oceanographic equipment compare to *Z*_SD_ data collected by trained scientists using the same methods? Unfortunately, it is impractical to take citizens and marine scientists to sea together to measure *Z*_SD_ at the same time and place to see how they compare. To overcome this impracticality, we compare *Z*_SD_ measurements collected by citizens and scientists using Secchi Disks to independent satellite estimates of *Z*_SD_, i.e. the comparison Secchi Disk measurements were not used to generate the satellite algorithm.

## Results

**Figure 1:**
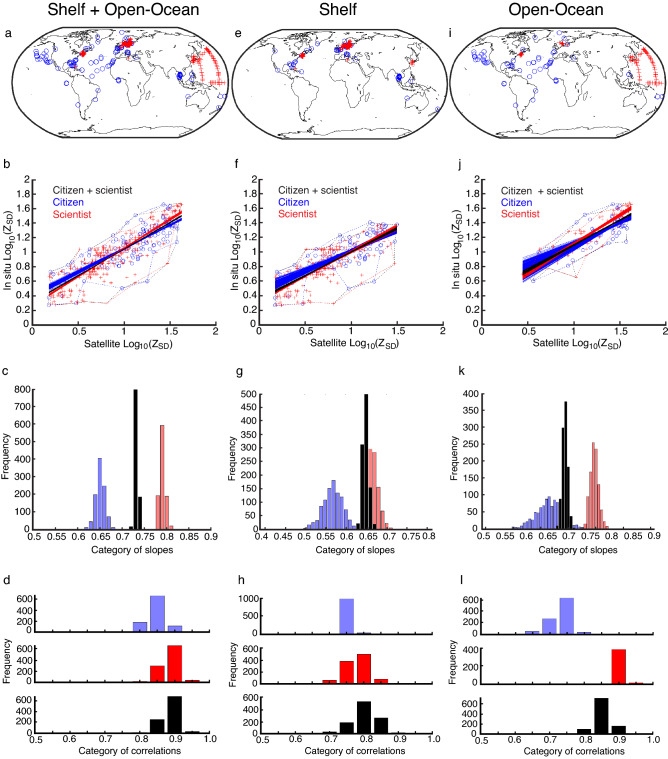
Relationships between scientist (red) and citizen (blue) log_10_(Z_SD_) and satellite log_10_(Z_SD_). (**a,e,i**) Maps showing the positions of the scientist and citizen Z_SD_ data for shelf + open-ocean, shelf, and open-ocean, respectively. Maps were created using MATLAB version 2021a, https://www.mathworks.com/products/matlab.html. (**b,f,j**) Scatterplots, polygons and regression lines showing the relationship between scientist, citizen, and satellite log_10_(Z_SD_) for shelf + open-ocean, shelf, and open-ocean data, respectively. Red and blue polygons delineate the scientist and citizen data, respectively, and regression lines in black represent combined scientist and citizen data. (**c,g,k**) Histograms of regression coefficients between scientist, citizen and scientist/citizen (black) and satellite log_10_(Z_SD_) data for shelf + open-ocean, shelf, and open-ocean, respectively. (**d,h,l**) Histograms of linear correlations between scientist, citizen, combined scientist and citizen (black), and satellite log_10_(Z_SD_) for shelf + open-ocean, shelf, and open-ocean data, respectively.

We found positive relationships between satellite *Z*_SD_ and both the scientist and citizen *Z*_SD_ (Fig. 1a–l). Removing 20 samples did not strongly alter the regression lines (Fig. 1b,f,j). The red and blue polygons delineating the scientist and citizen data, respectively, as well as the regression lines, overlapped. Although a Kruskal–Wallis test revealed the regression lines were significantly different between scientist and citizen programmes (p < 0.01), these differences in slope were small and so there was only a small slope alteration when the data were merged. Correlation analysis revealed that scientist correlations were slightly higher when ocean + shelf and shelf only were considered (Fig. 1d,h,l). The difference in correlation was higher when open-ocean data only were analysed (Fig. 1d,h,l). In general, scientific data had less variance in correlation except for the shelf where a higher variance may be explained by outliers (red crosses below the regression line within the red, dashed polygons) (Fig. 1b,f).

**Figure 2:**
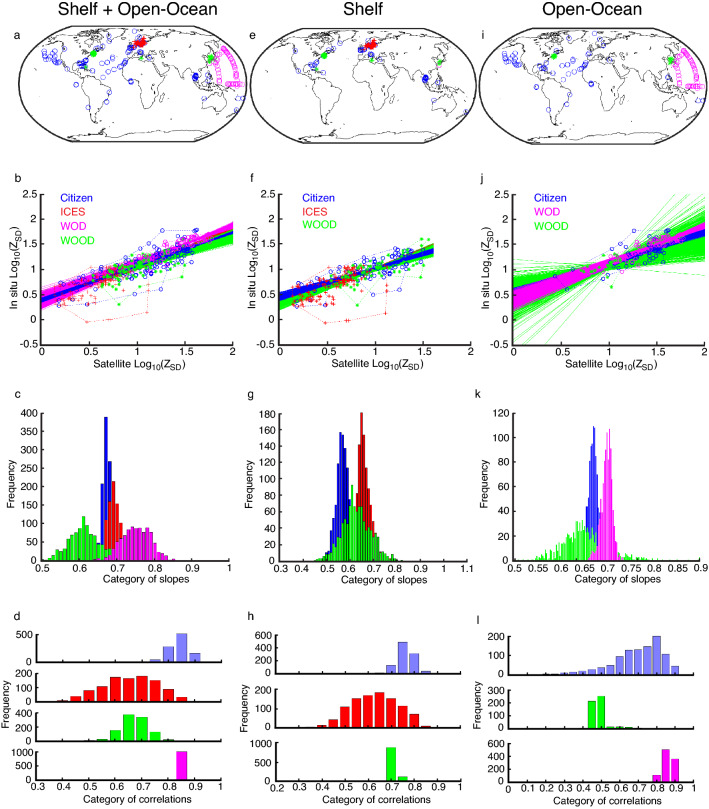
Relationships between WOD (magenta), WOOD (green), ICES (red) and citizen (blue) log_10_(Z_SD_) and satellite log_10_(Z_SD_). (**a,e,i**). Maps showing the positions of the WOD, WOOD, ICES, and citizen Z_SD_ data for shelf + open-ocean, shelf, and open-ocean, respectively. Maps were created using MATLAB version 2021a, https://www.mathworks.com/products/matlab.html. (**b,f,j**) Scatterplots, with red and blue polygons delineating the scientist and citizen data respectively, and regression lines showing the relationship between WOD, WOOD, ICES, citizen and satellite log_10_(Z_SD_) for shelf + open-ocean, shelf, and open-ocean, respectively. (**c,g,k**) Histograms of regression coefficients between WOD, WOOD, ICES, citizen and satellite log_10_(Z_SD_) for shelf + open-ocean, shelf, and open-ocean, respectively. (**d,h,l**) Histograms of the linear correlations between WOD, WOOD, ICES, citizen and satellite log_10_(Z_SD_) for shelf + open-ocean, shelf, and open-ocean, respectively.

**Figure 3:**
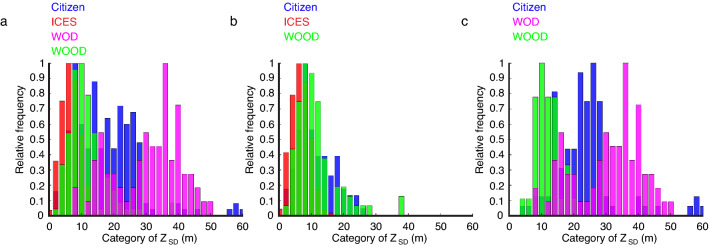
Histograms of *Z*_SD_ for the three scientific programmes and for citizens. (**a–c**) Histograms for WOD (magenta), WOOD (green), ICES (red) and citizen (blue) *Z*_SD_ data for shelf + open-ocean, shelf, and open-ocean, respectively.

Next, we analysed the data from WOOD, WOD, ICES and the citizens, separately (Fig. 2a–l). This analysis revealed that outliers could also be detected among scientific programmes (red crosses on the left below the regression line inside the red polygon, Fig. 2b,f). As before, all the regression lines were significantly different (Kruskal–Wallis test, p < 0.01). We noticed large variability in the regression lines, especially for WOOD when open-ocean data was considered; due to a low number of degrees of freedom. The histogram of the category of slopes showed a more significant separation and less overlap for all programmes in shelf seas than the open-ocean, which could reflect regional peculiarities (Fig. 2c,g,k). The histograms for each programme and for open-ocean + shelf, shelf and open-ocean (Fig. 3a–c) showed that part of the difference in regression coefficients may be due to location rather than the reliability of either citizen or scientist *Z*_SD_. We also found a difference in the correlations between open-ocean and shelf. With the exception of the open-ocean where citizen correlations were slightly lower than WOD, correlations involving citizen data were higher than other programmes (Fig. 2d,h,l).

**Figure 4:**
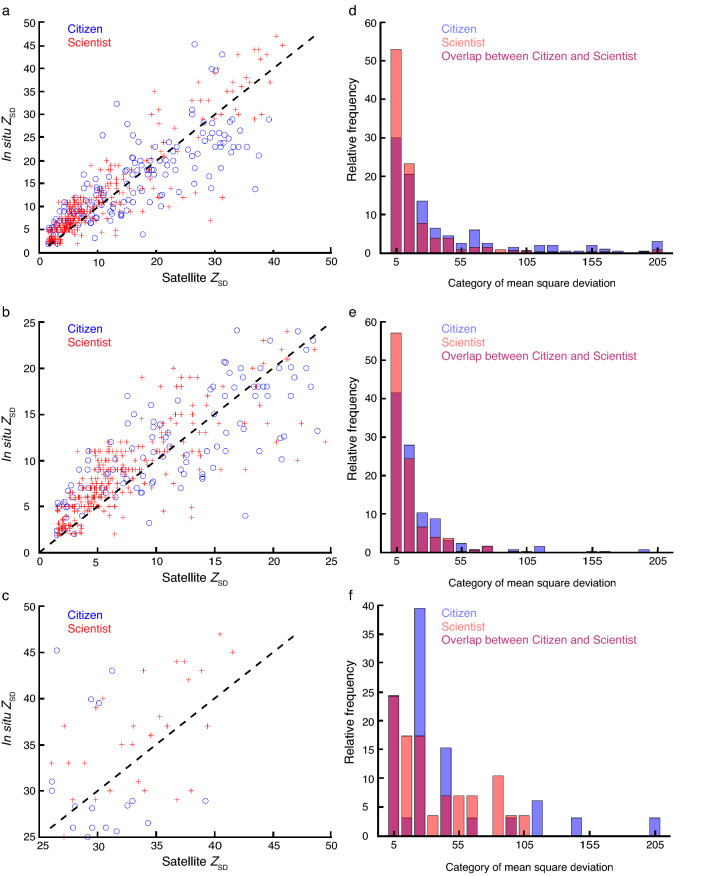
Relationships between citizen and scientist in situ* Z*_SD_, and satellite *Z*_SD_. (**a–c**) Scatterplots showing the relationship between scientist (red crosses), citizen (blue circles), and satellite *Z*_SD_ for all the whole dataset, where *Z*_SD_ < 25 m and where *Z*_SD_ ≥ 25 m, respectively. Black dashes represent the line y = x. (**d–f**) Histograms of the mean square deviation (m^2^) for the whole dataset, where *Z*_SD_ < 25 m and where *Z*_SD_ ≥ 25 m, respectively. Orange, blue and magenta bars represent scientist, citizen and where scientist and citizen data overlap, respectively.

When we compared the citizen and scientist data for systematic under- or overestimation using satellite *Z*_SD_ as a reference, scientists always highly overestimate satellite *Z*_SD_ as shown by the percentage of overestimates that are consistently well above 50% (Table 1, Fig. 4). We found that citizens tend to slightly underestimate satellite *Z*_SD_ when all data are considered (44.5%) and to a greater extent when *Z*_SD_ is greater than or equal to 25 m (36.4%). Both citizens and scientists overestimate *Z*_SD_ compared to satellite *Z*_SD_ when the measurement is less than 25 m. In all cases however, the under- or over estimation is less for citizens than for scientists. In contrast to the percentage of overestimates, the mean deviation (Table 1) is a more quantitative measurement of the degree of under- or overestimation and can be influenced by individual deviation (the individual differences between in situ and satellite *Z*_SD_ measurements). The mean deviation shows nearly the same results as the percentage of overestimates although when we consider all *Z*_SD_ together the underestimation by citizens (− 1.6 m) is slightly higher than the overestimation made by scientists (1.5 m) (Table 1). (The discrepancy observed between the percentage of overestimates and the mean deviation is likely due to a few large, individual deviations that influence the latter estimator). When we separated measurements of *Z*_SD_ below and above 25 m the mean deviation is smaller in absolute value for citizens in agreement with what is shown by the percentage of overestimates.

**Table 1:** Indirect comparison of citizen and scientist *Z*_SD_ data with respect to under and overestimation and closeness.

Analysis	Overestimate (%)	Mean deviation (m)	Mean square deviation (m^2^)
All citizen *Z*_SD_ data	44.5	− 1.6	39.2
All scientist *Z*_SD_ data	80.3	1.5	16.6
Citizen *Z*_SD_ data < 25 m	58.4	0.02	16.2
Scientist *Z*_SD_ data < 25 m	81.6	1.4	10.3
Citizen *Z*_SD_ data ≥ 25 m	36.4	− 1.3	39.8
Scientist *Z*_SD_ data ≥ 25 m	72.4	2.8	31.7

An underestimate of < 50% or a negative mean deviation means that in situ data underestimates satellite data and inversely. A percentage of overestimate or a mean deviation close to 0 means that there is no systematic under- or overestimation in the in situ data with respect to satellite data. When the mean square deviation is close to zero it means that in situ and satellite measurements are close together and exhibit a low variability.

The mean square deviation (here, a measure of the level of the closeness between in situ and satellite *Z*_SD_) revealed that *Z*_SD_ measured by scientists are consistently closer than *Z*_SD_ measured by citizens. Histograms prepared to examine the mean square deviation (Fig. 4d–f) suggest that a few observations made by citizens might cause the higher mean square deviation (Table 1). When we remove categories above 100 m^2^ the mean square deviation for citizens reduced by more than 50%, from 39.2 to 18.9 m^2^, and became very close to the scientist value (16.6 m^2^). Nevertheless, the degree of closeness between satellite and in situ* Z*_SD_ is higher for scientists than for citizens. When the first 3 categories of the histogram are considered together (Fig. 4d) they comprise 87% of the scientific data and 70% of the citizen data, which is a difference of 17%.

## Discussion

The Secchi Disk is among the simplest pieces of oceanographic equipment with its method of use, optical properties, limitations and variability well understood^18,30^; these features are taken into account in the instructions provided to citizens in the Smartphone application called Secchi that accompanies the citizen science study^17^. While our method of assessing citizen and scientist *Z*_SD_ data by comparison to satellite *Z*_SD_ is indirect and so imperfect (ideally citizens and scientists would go to sea together to make simultaneous measurements for such a comparison), our results show that scientists and citizens take reliable in situ measurements of *Z*_SD_ at sea.

Although we found that citizens tend to underestimate satellite *Z*_SD_, scientists tend to overestimate to a greater extent (Table 1) when compared to satellite *Z*_SD_. We do not know the cause of this under- and overestimation and cannot exclude this is a chance result, and because citizens and scientists under- and overestimate respectively, it is not possible at present to determine a correction factor; we nevertheless hope this can be resolved when more data is evaluated from both scientist and citizen programmes. We found higher variability in the mean square deviation for citizens and a 17% difference between citizen *Z*_SD_ and scientist *Z*_SD_ (when scientist data was combined from the three scientist datasets we used) (Table 1). When we performed the analysis on log_10_ transformed data the differences between citizens and scientists were much less apparent and to see them we need to look at the third digit of the mantissa (Supplementary Table S1).

The difference in the mean square deviation between citizens and scientists could be explained by several factors. For example, the three science programmes we used likely involve measurements collected by just a few individual scientists (for example on a single cruise), which will reduce the amount of individual observer variability that is inherent in the in situ measurement of *Z*_SD_^30^ when compared to the citizen data where many individual participants each collect a few measurements. Interestingly, when we excluded categories of mean square deviation above 100 m^2^ the closeness between citizens and scientists improved greatly. Consequently, as the citizen data set increases in size, we hope to be able to conduct analyses at a participant level to better understand individual variability and to remove outliers^31^. (The comparison between scientists and citizens may also change when data from many scientific programmes are considered and over longer time periods and larger spatial scales). Other influences on the data may include the fact that scientific measurements may be focused on a particular water body and so spatial heterogeneity will be different compared to the citizen data (Fig. 1a,e,i) and the fact that research vessels are likely to be more stable platforms compared to small boats and so measurements are likely to be easier to take. Finally, and importantly, scientists interact and train each other, which is different to a global citizen science study where similar training is impractical. Consequently, even though instructions are given to citizens in the Secchi smartphone application upon how to take *Z*_SD_ measurements, scientists are likely to show less variability because of their more formal training.

When we considered spatial heterogeneity, the *Z*_SD_ collected by citizens and scientists showed a better relationship to satellite *Z*_SD_ in the open-ocean (Fig. 2j,k). The poorer relationship found in shelf seas between in situ and satellite *Z*_SD_ (Figs. 1a,e,I, 2a,e,i) may therefore be due to the inherent properties of measurements made in specific oceanographic environments (Fig. 3) rather than the reliability of the Secchi Disk measurement in the hands of either citizens or scientists. Most likely, it is due to the greater spatial and temporal heterogeneity of shelf seas (where there may also be abiotic factors), which may all lead to an imperfect match between in situ and satellite *Z*_SD_ data due to violations in the assumptions underlying the satellite algorithm. The small overlap between volunteers (citizens) and professionals (scientists) (Fig. 1) when all the scientist data are combined together, compared to the clear overlap when we split the different science data sets (Fig. 2), may consequently, be explained by data originating from different ocean basins and environments, which is likely to alter in future when the data is more widespread.

Satellite estimates of *Z*_SD_ are influenced by the in situ* Z*_SD_ datasets used to generate the satellite algorithm and so will have inherent errors and biases. The Morel et al.^32^ algorithm we used is based upon a relationship between chlorophyll *a* and *Z*_SD_ for clear, open-ocean waters and a specific, scientist-collected, in situ* Z*_SD_ dataset where the majority of measurements were collected in the summertime in the northern hemisphere. Validation using MODIS Level-3 composite data and in situ* Z*_SD_ resulted in adjusted coefficients as the modelled values tended to exceed the measured values^32^, and it is these adjusted coefficients that have been used in the implementation, in other words, the algorithm was tuned to match the specific in situ* Z*_SD_ measurements.

In the open-ocean, satellite *Z*_SD_ can overestimate in situ* Z*_SD_ because the former assumes the layer extending down to *Z*_SD_ is homogenous. In contrast, chlorophyll *a* always varies with depth^32^; chlorophyll levels are typically lower at the surface than at depth under stratified open-ocean conditions and so satellite chlorophyll underestimates the depth-averaged value. Although a recent algorithm to calculate *Z*_SD_ from current satellite sensors has improved the relationship between satellite and in situ* Z*_SD_ data going forwards^33^, satellite remote sensing still only provides an integrated value that simplifies the biological and environmental vertical and horizontal heterogeneity^34,35^; as our results in shelf seas may indicate in the latter case in particular. Consequently, even in the modern era of remote sensing of ocean colour, in situ measurements of *Z*_SD_ provide an essential insight into ocean transparency and phytoplankton biomass.

Despite the growth in marine science^36^, due to the constraints of achieving widespread coverage by the limited scope of professional oceanographic cruises, the participation of the seafaring public provides an opportunity to extend oceanographic data collection. Citizen science has proven to contribute to gaps in our scientific knowledge in many fields^15,37^. By collecting ocean transparency data to contribute to historical and current in situ scientific *Z*_SD_ efforts, citizen scientists can help better understand the effects of current global-scale, climate-driven changes in the phytoplankton.

In order to assess long-term, global, ocean phytoplankton change in situ from the beginning of the 20th Century, without combining different types of data, it is necessary to increase the coverage of present-day Secchi Disk ocean transparency measurements to enable a direct comparison with historical Secchi Disk data^12,18^. Seafarers acting as citizen scientists provide a unique opportunity to collect in situ ocean transparency data. This study addressed the ninth principle of citizen science outlined by the ECSA, which recommends that citizen science should be evaluated for scientific data quality^26^. We found that when citizen and scientist *Z*_SD_ are compared to satellite *Z*_SD_ calculated using the algorithm of Morel et al.^32^ citizen *Z*_SD_ slightly underestimates the satellite value and scientist *Z*_SD_ deeply overestimates the value (Table 1). The percentage of estimates and the mean deviation suggest that citizens perform as well as scientists. Although citizen data is more variable, it remains reliable and consequently, with this understanding, it is possible to combine citizen-collected ocean transparency data together with scientific studies (Figs. 1d,h,l, 2d,h,l). We would recommend the type of analysis we have conducted when *Z*_SD_ data from different studies are combined so that long-term changes in phytoplankton biomass are estimated correctly.

## Methods

### Scientist measurements of Z_SD_

We obtained scientist *Z*_SD_ from 3 datasets. (1) The World Ocean Database (WOD) (https://www.nodc.noaa.gov/OC5/WOD/pr_wod.html)^38^. (2) The World-wide Ocean Optics Database (WOOD) (https://accession.nodc.noaa.gov/0092528)^39^. (3) The International Council for the Exploration of the Sea (ICES) (https://ocean.ices.dk/Project/SECCHI/)^22^. We used *Z*_SD_ data collected between 1997 to 2005, 1997 to 2001, and 1997 to 1998 from WOD, WOOD and ICES datasets, respectively.

### Citizen measurements of Z_SD_

We used measurements of *Z*_SD_ collected from 2013 to 2019 by seafarers participating in the on-going Secchi Disk study^17^.

### Satellite estimates of Z_SD_

Satellite estimates of *Z*_SD_ were the only possibility to compare citizen and scientist measurements of *Z*_SD_ since we do not have in situ data collected by citizens and scientists at the same locations contemporaneously. We obtained satellite measurements from the Hermes GlobColour website (http://hermes.acri.fr/index.php). The dataset, obtained by merging data from four sensors (SeaWiFS, MODIS, MERIS, VIIRS) with an arithmetic mean on a 4 km resolution spatial grid, provided overlapping time series of *Z*_SD_ for the time period 1997–2019. Estimates of *Z*_SD_ were calculated using the algorithm of Morel et al.^32^.

### Bathymetry and distance from the nearest coast

We only used measurements of *Z*_SD_ collected in water > 25 m deep and > 1 km from the coast. Bathymetry data were obtained from a global ocean bathymetry chart (0.1 degrees latitude by 0.1 degrees longitude)^40^. We calculated distance from the nearest coast from a global data set of distances from the nearest coastline estimated at a spatial resolution of 0.01 degrees^41^. We used the nearest method of interpolation^42,43^ to give a bathymetry or distance from the nearest coast for each *Z*_SD_.

### Data pre-processing

We attributed a value of satellite-based *Z*_SD_ for each in situ measurement of *Z*_SD_ if a satellite estimate was available within 100 km of the in situ sample, plus three days before or after it’s measurement. To retain as many measurements as possible near to the coast we calculated the estimation using the nearest method of interpolation^43,44^.

### Data analyses

We conducted three analyses: (i) indirectly comparing all scientist *Z*_SD_ data together and citizen *Z*_SD_ data to satellite *Z*_SD_ (ii) comparing the three scientific programmes’ data separately and citizen data, to satellite *Z*_SD_ and (iii)  determining whether any biases occurred between citizen and scientist *Z*_SD_ data when compared to satellite data.

First, we examined the relationship between satellite *Z*_SD_ and both scientific (all programmes combined) and citizen *Z*_SD_ data using scatter plots for open-ocean and shelf systems; shelf where bathymetry ≤ 200 m and distance to coast > 1 km, and open-ocean where bathymetry > 200 m. We highlighted the datasets by drawing polygons around only-scientist, only-citizen and both scientist/citizen data. We performed three regression analyses^37^ (i) all data (scientist/citizen), (ii) scientist and, (iii) citizen. Each regression analysis was performed 1000 times, each time removing 20 samples to examine potential outliers’ influence on the regression. Increasing the removal of samples did not alter our conclusions; the variance increased when the removal of samples was higher and conversely. We therefore created 1000 regression lines for each of the three regression analyses. We displayed the histograms of all regression coefficients for scientist, citizen and scientist/citizen data. A Kruskal–Wallis test was applied to test whether regression coefficients for each dataset were significant.

We also calculated the linear correlations between both scientist and citizen *Z*_SD_ and satellite *Z*_SD_. We performed 1000 simulations for this analysis, recalculating the correlation after randomly choosing 100 pairs of points when all areas (shelf + open-ocean) were selected, 100 for just shelf data and 90 for open-ocean data. The number of pairs of points available drove the choice of these thresholds. We used histograms to display the correlation coefficients.

Second, we performed similar tests to investigate the three scientific programmes separately (WOD, WOOD and ICES) and citizen data. However, while we used 1000 simulations to calculate linear correlations, we considered 65 pairs of points for open-ocean + shelf data and shelf data, and 29 pairs of points for open-ocean. The ICES and Pacific Ocean data were not used when we tested the relationships between scientist *Z*_SD_ data and satellite *Z*_SD_ for the open-ocean and shelf, respectively. We also produced histograms of *Z*_SD_ for WOD, WOOD, ICES and citizen data for open-ocean + shelf, shelf, and open-ocean to determine if any changes in slopes (regression coefficients) might be explained by the sampling region.

Third, we determined if any biases occurred between citizen and science data when each was compared to satellite measurements independently. Specifically, we investigated whether citizen or scientist in situ measurements of *Z*_SD_ underestimated or overestimated satellite measurements of *Z*_SD_ that were derived from the algorithm of Morel et al.^32^,*Z*_SD_ (i.e. without log_10_ transformation). Although a logarithmic transformation improves the regression analysis by stabilising the variance of the variable (homoscedasticity) (Fig. 1b), it was more straightforward to compare real values; we present the results for a logarithmic transformation in Supplementary Table S1. We assessed three estimators that are generally used to examine the performance of diversity indices^45^: (i) the percentage of overestimates, (ii) the mean deviation MD and (iii) the mean square deviation (MSD).

The percentage of overestimates is a measure of bias varying between 0 and 100%. An unbiased estimator should have a value of 50%, meaning that it underestimates or overestimates 50% of the time. In our context, we use this estimator to determine how frequently in situ citizen or scientist measurements were above or below satellite estimates. For example, a value of 20% for citizen *Z*_SD_ would suggest that citizens deeply underestimate *Z*_SD_. The MD (expressed here in m) is the average of the differences between an estimator and its true or expected value. In the context of our study, it is the mean difference between in situ and satellite measurements and it is calculated as follows:

$$MD=\frac{\sum_{i=1}^{n}\left({O}_{i}-{S}_{i}\right)}{n},$$

where *O* is the in situ* Z*_SD_ measurement (either citizen or scientist), *S* is the satellite measurement of *Z*_SD_ and *n* is the number of couples of points (for example, citizen and satellite estimates of *Z*_SD_). MD can therefore be negative if in situ values are lower than satellite estimates and inversely.

In contrast to the two previous estimators, MSD is used generally to estimate accur^45^. It is calculated by estimating the average of the square of the differences between in situ and satellite measurements as follows:

$$MSD=\frac{\sum_{i=1}^{n}{\left({O}_{i}-{S}_{i}\right)}^{2}}{n}.$$

MSD (expressed here in m^2^) varies between 0 and $\infty$ and is a measure of closeness between in situ and satellite data. A value close to 0 indicates that the accuracy of the estimator is high and inversely. In our case, it suggests that the values originating from in situ measurement (either citizen or scientist) are constantly close to the values assessed from satellites using the algorithm of Morel et al.^32^.

We also calculated histograms of the deviations (a deviation is the difference between *O* and *S*) between in situ and satellite measurements to compare in greater detail the degree of accuracy between citizens and scientists.

The estimators and histograms, were calculated (i) on the whole set of untransformed data (see Fig. 4a) and data (ii) where *Z*_SD_ < 25 m and (iii) where *Z*_SD_ ≥ 25 m. They were also calculated between in situ citizen and scientist data. Therefore, we had 6 values of percentages of overestimates, MD and MSD as well as six histograms (displayed as two per figure) at the end of the procedure (see Table 1, Fig. 4).

## Supplementary Information

Supplementary Table S1.
