# Supplementary material for: Citizens and scientists collect comparable oceanographic data: measurements of ocean transparency from the Secchi Disk study and science programmes
Source: Sci Rep. 2021 Jul 29;11:15499. doi: 10.1038/s41598-021-95029-z (PMC8322096; doi:10.1038/s41598-021-95029-z)
Supplement: Supplementary file 1 — Supplementary Table S1. [file 41598_2021_95029_MOESM1_ESM.docx]

Supplementary Table S1. Indirect comparison of citizen and scientist log_10_ transformed *Z*_SD_ data with respect to under and overestimation and closeness. An underestimate of <50% or a negative mean deviation means that *in situ* data underestimates satellite data and inversely. A percentage of overestimate or a mean deviation close to 0 means that there is no systematic under- or overestimation in the *in situ* data with respect to satellite data. When the mean square deviation is close to zero it means that *in situ* and satellite measurements are close together and exhibit a low variability.

| Analysis | Overestimate  (%) | Mean deviation  (m) | Mean square deviation  (m^2^) |
| --- | --- | --- | --- |
| All citizen log_10_ *Z*_SD_ data | 44.5 | -0.006 | 0.033 |
| All scientist log_10_ *Z*_SD_ data | 80.3 | 0.094 | 0.034 |
| Citizen log_10_ *Z*_SD_ data < 25 m | 58.4 | 0.034 | 0.039 |
| Scientist log_10_ *Z*_SD_ data < 25 m | 81.6 | 0.104 | 0.036 |
| Citizen log_10_ *Z*_SD_ data ≥ 25 m | 36.4 | -0.023 | 0.007 |
| Scientist log_10_ *Z*_SD_ data ≥ 25 m | 72.4 | 0.033 | 0.005 |
